# Supplementary material for: An updated systematic review of the risk factors for unplanned dialysis initiation
Source: Clin Kidney J. 2024 Oct 29;17(12):sfae333. doi: 10.1093/ckj/sfae333 (PMC11635165; doi:10.1093/ckj/sfae333)
Supplement: sfae333_Supplemental_Files [file sfae333_supplemental_files.zip › 824.docx]

An updated systematic review of the risk factors for unplanned dialysis initiation

Winnie Magadi^1, 2^

Kate Birnie^2^

Shalini Santhakumaran^1^

Fergus J. Caskey^2^

Yoav Ben-Shlomo^2^

^1^ UK Renal Registry, UK Kidney Association, Bristol, UK

^2^ Department of Population Health Sciences, Bristol Medical School, University of Bristol, Bristol, UK

**Correspondence to:**

Winnie Magadi

E-mail: Winnie.Magadi@bristol.ac.uk

**Abbreviations:**

AKI Acute Kidney Injury

CKD Chronic Kidney Disease

CVC Central Venous Catheter

DA Dialysis Access

EDS Emergency Dialysis Start

EGFR Estimated Glomerular Filtration Rate

ESA Erythropoietin Stimulating Agent

ESKD End Stage Kidney Disease

GP General Practitioner

HD Haemodialysis

ICA Iodinated Contrast Agents

NOS Newcastle Ottawa Scale

NSAIDs Non-steroidal Anti-infammatory Drugs

PD Peritoneal Dialysis

PPI Proton Pump Inhibitors

PRD Primary Renal Diagnosis

PRISMA Preferred Reporting Items for Systematic Review and Meta-Analysis

RASB Renin-angiotensin System Blocker

KRT Kidney Replacement Therapy

SCr Serum Creatinine

UDI Unplanned Dialysis Initiation

UDS Urgent Dialysis Start

**GRAPHICAL ABSTRACT**

**
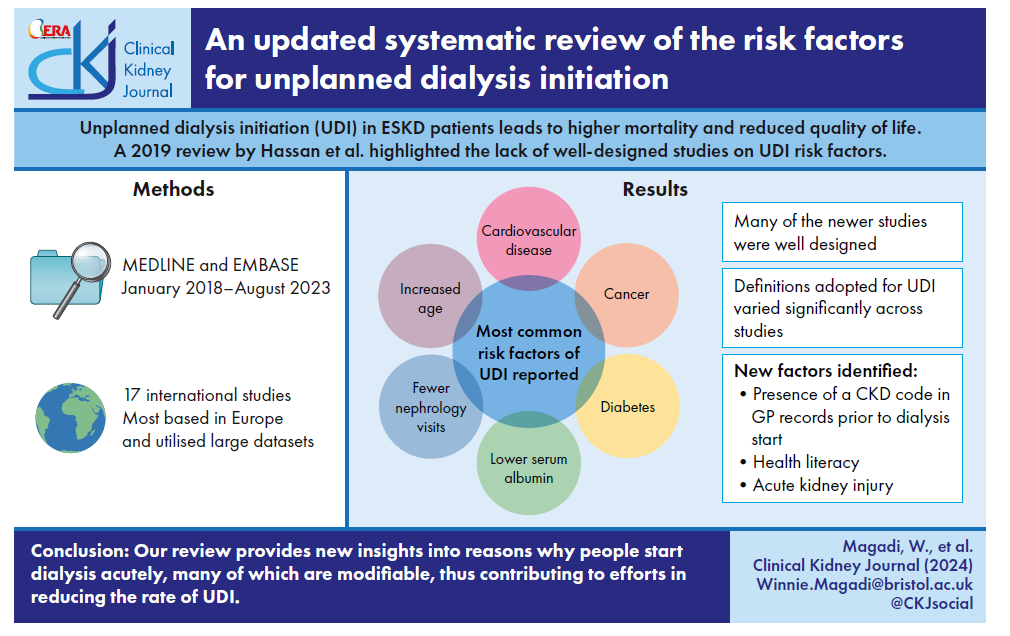
**

**ABSTRACT**

**Background.**

Previously, a comprehensive review of the risk factors for unplanned dialysis initiation (UDI) was conducted by Hassan et al. (2019), based on studies published up to end of 2017. They demonstrated that high quality data and well-designed studies on the subject are lacking. Thus, we updated their review to establish the modifiable factors associated with UDI.

**Methods.**

MEDLINE and EMBASE were searched from January 2018 to August 2023. Following several rounds of screening, we identified 17 international studies (majority of which were based in Europe) that met the eligibility criteria.

**Results.**

Many of the included studies were well designed, utilised very large datasets, and adopted multivariate analyses to examine associations between patient characteristics and UDI. Definitions of UDI varied across studies, i.e. timeliness of presentation, vascular access type, initiating dialysis as an inpatient/outpatient, or for life threatening indications.

The most common risk factors reported were cardiovascular disease, increased age, cause of kidney disease, cancer, diabetes, lower serum albumin, increased rate of estimated glomerular filtration rate (eGFR) decline and fewer number of nephrology visits prior to dialysis start. These were in line with those reported by Hassan et al. However, our updated review revealed several other important predictors of UDI, i.e. worse coding of chronic kidney disease (CKD) in the GP health record, lower health literacy, and having an acute kidney injury (AKI).

**Conclusions.**

Our review provides new insights into reasons why people start dialysis in an unplanned manner, many of which are modifiable, thus contributing to efforts in reducing the rate of UDI.

**KEY LEARNING POINTS**

**What was known:**

- The incidence of unplanned dialysis initiation (UDI) is reported to be high globally, ranging from 15-60%. Patients who start dialysis in an unplanned manner experience worse clinical outcomes than those who have planned care. Factors such as presenting late to a nephrologist are known to be associated with UDI.
- Previously, a comprehensive review on the risk factors for UDI was conducted by Hassan et al. (2019) and demonstrated that well designed studies on the subject are limited. The varying definitions of UDI adopted across studies also makes it difficult to draw meaningful conclusions.
- There is a need to identify the modifiable risk factors for UDI in the literature, based on up-to-date studies utilising properly adjusted analyses. This will aid improvements in the care and outcomes for patients with advanced CKD transitioning to dialysis.

**This study adds:**

- Our updated review highlights several novel risk factors for UDI that have not received much attention in the literature. These include social factors i.e. living alone and reduced mobility, as well as factors related to nephrology care i.e. worse CKD coding prior to start of kidney replacement therapy (KRT).
- The inclusion of well-designed studies, utilising rich and large datasets to examine the associations at hand, strengthens the evidence on the commonly reported risk factors for UDI. Given that some of the factors are modifiable, our study contributes to efforts in reducing the rate of UDI in the population.
- The issue of inconsistent definitions of UDI persists making comparisons between studies difficult. Further, given the differences in clinical practices across the world in relation to how and when patients begin dialysis treatment, tackling this issue of UDI among CKD patients remains a complex one.

**Potential impact:**

- Our review suggests a need to further investigate specific modifiable factors related to nephrology care that are associated with UDI. The evidence that worse CKD coding in GP records increases one’s risk of UDI could be used to guide clinical practice by encouraging better surveillance of CKD patients.

**Keywords:** mortality risk, systematic review, unplanned dialysis initiation

**BACKGROUND**

It is evident that how a patient with chronic kidney disease (CKD) who transitions to end-stage kidney disease (ESKD) is managed can significantly impact their clinical outcomes [1]. For individuals that commence treatment for ESKD, a number of factors such as early presentation to a nephrologist, patient education and better coordination of medical care are likely to lead to planned dialysis initiation [2]. However, rates of unplanned dialysis initiation (UDI) remain high, with estimates in United Kingdom and North America reported to be between 40 – 60% [3]. This is despite countries publishing guidelines on the management of patients with CKD, with clear specifications on pre-dialysis care pathways [4]. Patient trajectories for those starting dialysis treatment differ across countries given the varying health practices adopted i.e., referral criteria tend to reflect the structure of the healthcare system and availability of resources and services [4] . Nevertheless, certain factors including presence of multidisciplinary teams and shared decision are recognised as being internationally relevant and often reflected in the guidelines for the management of patients with CKD transitioning into dialysis [7].

Determining the risk factors for UDI is important given that outcomes and experiences of care for patients who start treatment in this manner are significantly worse than for those who have planned care [8, 9]. A reduced quality of life, more frequent and longer hospitalisations, increased patient morbidity and mortality, as well as higher healthcare costs are all associated with UDI[10-12]. Previous studies have shown that having a planned dialysis start (as opposed to an early referral to a nephrologist) is what improves both the short- and long-term health-related quality of life [13]. However, one of the major challenges in identifying the contributing risk factors for UDI is the lack of consensus in the definitions adopted. These range from time between referral to a nephrologist and initiation of dialysis to the setting in which patients initiate dialysis i.e. under ‘emergency’ conditions or starting dialysis an inpatient versus outpatient [3]. Previously, a comprehensive review of the risk factors for unplanned dialysis initiation (UDI) was conducted by Hassan et al. in 2019, and comprised data up to end of 2017 [3]. They found that the most common risk factors reported included increased age, cardiovascular disease, lower serum albumin and late referral. However, the current evidence on the modifiable factors contributing to the high rate of UDI is inconclusive [14]. While early referral to a nephrologist is associated with lower mortality after dialysis initiation, unplanned dialysis (and the poor outcomes associated with it) occurs in patients both known and unknown to nephrology services and in early and late referrals [15, 16]. The review by Hassan et al. also highlighted the lack of well-designed studies examining the contributing risk factors as these were largely based on descriptive, unadjusted analyses; utilising data collected several years ago which may not be reflective of the current clinical practice patterns.

Thus, we set out to conduct a systematic review to identify the risk factors for UDI, based on the new evidence from 2018-2023. Our primary objective was to identify the modifiable factors associated with unplanned dialysis starts, as well as any other factors that were not reported by Hassan et al. in their review. The secondary objective was to conduct meta-analyses on the identified risk factors by combining the results published by Hassan et al. with those in this updated review. We hypothesize that the more recent studies will be better designed and utilise better quality data to examine the determinants of UDI.

**METHODS**

**Search strategy and data extraction**

A systematic review was conducted in accordance with recommendations from the Preferred Reporting Items for Systematic Review and Meta-Analysis (PRISMA) 2020 statement[17]. We utilised and adapted the search strategy developed by Hassan et al. (2019) [3] (see appendix A), limiting the search to studies published from 1^st^ January 2018 to 23^rd^ August 2023. The initial review covered the period from inception to February 2018, although the last published study reported in 2017, therefore only newer studies that were not captured in the previous review were included. A systematic search of the following electronic databases was conducted by WM with the assistance of a medical librarian (Sarah Herring) experienced in systematic reviews to identify potentially eligible published papers: Ovid MEDLINE and Epub Ahead of Print, In-Process, In-Data-Review & Other Non-Indexed Citations and Daily and EMBASE. The identified studies from the search were then screened for relevance by WM, beginning with their titles, abstracts, and then full text. To determine whether any relevant articles had been missed, two reviewers (SS and KB) screened 10% of the identified studies in the first round, with any disagreements being resolved by discussion. The qualification of the searchers was: WM MSc, KB PhD, and SS PhD. Lastly, data on the study characteristics, author details, methods, interventions, definition of the outcome, and results, were then extracted from all eligible studies identified in the review and recorded on a spreadsheet (see appendix B).

**Eligibility criteria**

We included any observational study which examined any potential risk factor (due to our review being exploratory in nature) for UDI. Eligible studies were those that reported criteria to define UDI and characteristics of patients who had an unplanned versus planned dialysis initiation. As there is currently no agreed definition of UDI in the literature, we included studies that described whether patients started dialysis in a planned or unplanned manner e.g. based on hospitalisation status or type of vascular access used. We included studies of patients of any age (although those based solely on children were excluded) with CKD and/or receiving dialysis (haemodialysis or peritoneal dialysis). Participants with CKD were defined as individuals with an estimated glomerular filtration rate (eGFR) <60 ml/min/1.73 m2 over 3 months or more, irrespective of cause, in accordance with the published definitions of the National Kidney Foundation Kidney Disease Outcomes Quality Initiative (KDIQI) [18]. Studies were also required to be available in full-text and published in a peer-reviewed journal in the English language. We excluded non-English articles, studies published only in abstract form, case reports, narrative reviews, and editorials.

**Assessment of study quality**

To assess the quality of the included studies, a modified version of the Newcastle Ottawa scale (NOS) was used[19]. Studies were given a maximum score of 5 depending on whether they met multiple criteria. For example, studies that were properly adjusted based i.e. conducting multivariate analyses to examine the question at hand and controlled for the relevant confounders received a higher score (2 points maximum). The outcome domain was removed from the NOS, as we did not examine outcomes associated with unplanned dialysis. Further, we assessed how representative the study cohorts were of the population of interest at risk we were trying to capture i.e. those with advanced CKD (3 points maximum). It is worth noting that the definition used in each study influenced what study population it made sense to restrict to, especially in relation to age.

**Data synthesis**

Our systematic review was exploratory in nature; therefore, it was not possible to know how consistently the risk factors, characteristics, or outcomes would be reported across studies. Thus, we planned to produce a descriptive report of the results for each of the identified risk factors and outcome in cases where pooling the data was not feasible due to inadequate information or excessive heterogeneity. Where data permitted, we performed meta-analyses of the odds ratios for the most common risk factors for UDI to obtain pooled estimates of the association with the outcome. This was dependent on whether there were at least 10 studies identified after combining the results from Hassan et al.’s review (48 in total) with those identified in this updated review. We utilised a random-effects inverse-variance model as these allow for anticipated heterogeneity between studies. Heterogeneity may be due to variation in the definition of unplanned dialysis start; variation in the study characteristics such as age of patients, study setting and location, sample size, and so forth. This was performed using the metan command in Stata 18 [20]. To evaluate the percentage of variation between studies that cannot be attributed to within-study variation we examined the I² value. Forest plots were used to display results of the meta-analyses. Where it was not possible to pool data across studies due to inadequate information or excessive heterogeneity, we provided a descriptive summary of the results in a table format only. Further, we planned to conduct additional analyses to evaluate potential sources of heterogeneity between studies where 10 or more studies were available for a particular risk factor.

**Results**

Figure 1 outlines the study selection process for the current review. In total, there were 3,678 studies identified from the search using the two databases. After screening and removal of duplicates, 17 studies (with 13 unique cohorts) were found to meet the eligibility criteria.

**Figure 1: A flowchart of the selection process and identification of newer studies in the updated review^[[1]](#footnote-1)^**


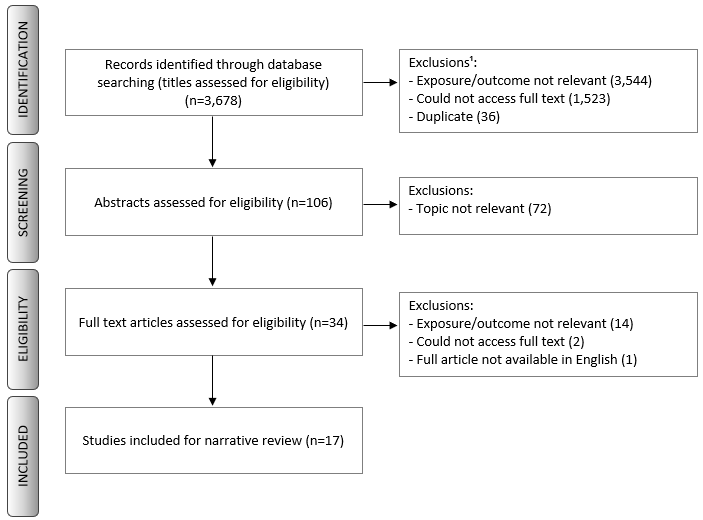


**Patient and study characteristics of newly identified studies**

Fourteen of the seventeen included studies examining the risk factors for UDI were retrospective in nature, with the remaining three either adopting a prospective design (2) or an ecological study design (1). Most of the studies were based in Europe i.e. England (2), France (7), Italy (1), Nordic and Baltic countries (1), while the remaining 6 were in North and South America, Asia and Australia. Our review identified single-centre and multi-centre studies, with the latter having much larger cohorts of patients i.e., data obtained from national registries or gathered from several nephrology clinics. While majority of the studies were based on adult patients, two of the studies included children (under 16 years) as well. One study limited its population to the elderly (defined as 65 years and over), and the two studies based in England consisted of younger and more ethnically diverse populations. In addition, two of the included studies conducted in Brazil limited their analyses to patients on peritoneal dialysis (PD). The remaining studies were based either on patients initiating haemodialysis (HD), any dialysis treatment or chronic kidney disease patients for the prospective studies. Lastly, 6 studies excluded patients with an acute kidney injury (AKI) who required dialysis, and 6 studies excluded patients with a pre-emptive transplantation.

Regarding the quality of included studies, the results of the modified NOS are summarized in Supplementary Table 1. Majority of the studies had a score of 4 or above (out of a maximum of 5; range 1-5) in relation to cohort selection and comparability. Only one study scored a maximum of 5 points, and this was due to the remaining studies mainly losing a point for their retrospective design. However, a large proportion of the studies obtained the full score for comparability i.e. adjusted for the relevant factors associated with UDI.

**Definitions for Unplanned Dialysis**

The definitions adopted for UDI varied significantly across studies. The proportion in each category were as follows: Vascular access used for first dialysis – 41%; Started dialysis for life threatening indications – 41%; Timeliness of referral/ presentation, +/- attendance at multidisciplinary clinic – 12%; Started dialysis as inpatient or outpatient – 6%. More detailed descriptions are provided in table 1.

**Table 1: Definitions of unplanned dialysis initiation**

| **Study** | **Definition** |
| --- | --- |
| Michel et al., 2018 [21]  Padilla et al., 2019 [22]  Raffray et al., 2020 [5]  Fages et al., 2021 [23]  Pétureau et al. 2021 [24] | Emergency start was defined as a first dialysis session within 24 hours after a nephrology visit, for life threatening conditions, including acute pulmonary edema, severe hyperkalaemia or acidosis, uremic confusion, or pericarditis. |
| Lee et al., 2018 [25] | Central venous catheter (CVC) use was defined as an unplanned dialysis start. |
| Arulkumaran et al., 2019 [12] | Unplanned starters were patients known to renal services for >90 days who required acute initiation of renal replacement therapy. Urgent starters were those patients known to renal services <90 days who required acute renal replacement. |
| Olaitan et al., 2019 [8] | Unplanned dialysis was described as receiving <90 days follow-up by a nephrologist in a dedicated, multidisciplinary clinic for patients with an estimated glomerular filtration rate (eGFR). |
| Shimizu et al., 2020 [26] | Patients who started unplanned haemodialysis therapy used a short-term CVC in every case, because they either could not wait for the placement of catheters or the maturation of the vascular access. |
| Heaf et al. 2021 [27] | Dialysis initiation was classified as suboptimal if: (i) the access for HD was a temporary vascular catheter; (ii) the access for HD was a tunnelled catheter, but a later AVF/AVG was planned; and (iii) the access was a peritoneal catheter, and peritoneal dialysis (PD) was started <6 days after placement, due to dialysis requirement before the catheter had completely healed. |
| Silva et al. 2021 [28] | Patients were placed on the urgent-start PD group if they had an urgent indication of KRT and started PD within 72 hours after catheter insertion or early-start PD group if PD initiated between 3 and 14 days. Patients that, for any reason, needed HD previously to PD start were also considered as early-start. |
| Chung et al. 2021 [29] | Suboptimal dialysis initiation was defined as having a CVC used at haemodialysis start. |
| Tazza et al. 2021 [30] | Unplanned dialysis started was defined as access at the start via a CVC, both tunnelled or non-tunnelled or AVF. |
| Nakayama et al. 2022 [31] | UDI was defined as urgent hospitalization to start dialysis or the unexpected initiation of dialysis while hospitalized for any purpose other than dialysis initiation; all other instances were considered planned dialysis initiation. |
| Takayama et al. 2022 [32] | Emergent haemodialysis initiation was defined as haemodialysis initiation with a temporary vascular catheter without elective permanent vascular access, or unplanned haemodialysis initiation due to the need for critical care regardless of elective permanent vascular access. |
| Pilatti et al. 2022 [33] | The urgent-start PD group consisted of patients that had an indication for urgent dialysis initiation, started PD within 7 days after Tenckhoff catheter implantation, and did not receive HD prior to PD. |
| Tachikart et al. 2023 [34] | In addition to the REIN registry definition of urgent start dialysis (USD), the authors developed another classification with 3 groups of patients defined as follows: – USD: defined as the absence of prior creation of a dialysis access (DA) and no creation planned within a period of a month when the patient starts dialysis. – Unplanned non-urgent start dialysis corresponds to the following situations:  – Patients who start with a functional DA but recently created that is to say: less than 4 weeks before dialysis start for a native arteriovenous fistula, 2 weeks for prosthetic fistula and 1 week for a PD catheter.  – Patients who had the creation of DA but which is not functional at dialysis start because of complication and consequently require a CVC.  – Patients who start with a CVC while waiting for a kidney transplant (with deceased or living donor) and have no project of other DA because the period of dialysis is expected to be short. |

**Risk factors associated with UDI in the newly identified studies**

Table 2 highlights the risk factors in each of the included studies that were associated with UDI, as well as the direction of the association. The most commonly reported predictors of UDI were cardiovascular disease, increased age, cause of kidney disease, BMI, cancer, diabetes, lower serum albumin, rate of eGFR decline and fewer nephrology visits prior to dialysis initiation. Interestingly, the effects of age, sex and hypertension on UDI differed across studies. From our analysis, we also identified factors that were not reported by Hassan et al. (see *Added* category). These included social factors, medications and health conditions i.e. living alone, living in urban areas, stable inhabitants, low health literacy, being treated in a public hospital (versus private clinic), reduced mobility, number of physical disabilities, winter season, presence of liver or respiratory disease, presence of AKI, potassium binders, cachexia, exposure to hyper polypharmacy, NSAID use and ionized calcium use. Several factors related to nephrology care were also identified: reduced creatinine monitoring, fewer nephrology related hospital stays, less KRT preparation, worse CKD coding prior to RRT and fewer number of General Practitioner (GP) consultations in the year prior to RRT, Hepatitis B vaccination ever prior to RRT, prescribed statins in the 6 months prior to RRT.

**Table 2: Risk factors associated with unplanned dialysis initiation (UDI).**

🡩 Number of studies finding evidence that the variable had a higher risk of UDI.

– Number of studies where there was no evidence of an association with UDI.

🡫 Number of studies finding evidence that the variable had a lower risk of UDI.

|  | **Variable** | **No. studies** | | | **Total sig.** | **Notes** |
| --- | --- | --- | --- | --- | --- | --- |
|  |  | **🡩** | **–** | **🡫** |  |  |
| **Demographics** | Age | 3 | 9 | 2 | **5** | *Two studies found that younger age was associated with UDI, while the remaining three studies showed an association between increased age and UDI.* |
|  | Sex (Male) | 2 | 11 | 1 | **3** | *Two studies showed that male sex is associated with a higher risk of UDI, while one study found that females had the higher risk.* |
|  | Low SES and rural residence | 1 | 0 | 0 | **1** |  |
| **Comorbidities** | Cardiovascular disease | 10 | 1 | 0 | **10** |  |
|  | Cause of kidney disease | 5 | 0 | 0 | **5** | *Study 1: The risk of UDI was higher in patients with renovascular disease or 'other' compared to diabetic nephropathy. Study 2: Those with polycystic renal disease were less likely to have an unplanned start than those with other/uncertain PRD. Study 3: Those with hypertensive and vascular nephropathy, and glomerulonephritis were less likely to initiate UDI, and those with 'other' PRD were more likely. Study 4: Patients with renal vascular disease and cystic renal disease/familial nephropathy were less likely to start unplanned dialysis compared with those with unknown PRD, while those with systemic disease were more likely to than the comparator group. Study 5: Acute nephropathy was associated with an unplanned start compared with slow progressive disease.* |
|  | Diabetes | 4 | 8 | 0 | **4** |  |
|  | Cancer | 4 | 3 | 0 | **4** |  |
|  | BMI/Weight | 0 | 2 | 4 | **4** | *Those with a higher BMI were less likely to initiate an unplanned dialysis start.* |
|  | COPD | 3 | 1 | 0 | **3** |  |
|  | Other comorbidity score | 2 | 0 | 0 | **2** | *A higher count of long-term health conditions (excluding CKD) was associated with UDI.* |
|  | Higher Bp/Hypertension | 1 | 4 | 1 | **2** | *One study found that hypertensives are more likely to have a UDI, and the other found that they are less likely.* |
|  | Charlson comorbidity index | 1 | 0 | 0 | **1** |  |
|  | Peripheral vascular disease | 1 | 2 | 0 | **1** |  |
|  | Smoking | 1 | 2 | 0 | **1** |  |
|  | Polycystic kidney disease | 0 | 0 | 1 | **1** |  |
| **Biochemistry** | Serum Albumin | 4 | 1 | 0 | **4** | *Lower serum albumin was associated with a higher risk of UDI.* |
|  | Haemoglobin | 2 | 1 | 0 | **2** | *Lower Hb levels were associated with a higher risk of UDI.* |
|  | Urea | 1 | 0 | 0 | **1** | *Increased levels of urea were linked to UDI.* |
| **Nephrology care** | Number of nephrology visits | 4 | 1 | 0 | **4** | *UDI was associated with fewer nephrology visits prior to starting dialysis.* |
|  | Rate of eGFR decline | 4 | 0 | 0 | **4** | *A faster decline in kidney function was associated with a higher risk of UDI.* |
|  | Late referral | 2 | 0 | 0 | **2** |  |
|  | eGFR at first visit | 1 | 0 | 0 | **1** |  |
|  | Time from KRT discussion to dialysis start | 1 | 0 | 0 | **1** | *Less discussion and planning around KRT prior to dialysis start increases one’s risk of UDI.* |
|  | Time known to nephrology | 1 | 0 | 0 | **1** | *Less time known to nephrology services was associated with UDI.* |
| **Medications** | Renin angiotensin (RAS) inhibitors | 0 | 0 | 1 | **1** | *The use of RAS inhibitors is significantly associated with a lower incidence of UDI.* |
| **Added** | Respiratory disease | 3 | 1 | 0 | **3** |  |
|  | Mobility | 3 | 0 | 0 | **3** | *Those who are less mobile are more likely to have an unplanned start.* |
|  | Liver disease | 3 | 1 | 0 | **3** |  |
|  | Acute kidney injury | 2 | 0 | 0 | **2** |  |
|  | Potassium binders | 0 | 0 | 1 | **1** |  |
|  | Winter season (versus summer) | 1 | 0 | 0 | **1** | *Emergent haemodialysis initiation was significantly more frequent in the winter than in the remaining seasons.* |
|  | Living alone | 1 | 0 | 0 | **1** |  |
|  | Low health literacy | 1 | 0 | 0 | **1** |  |
|  | Cachexia | 1 | 0 | 0 | **1** |  |
|  | NSAID use | 1 | 0 | 0 | **1** |  |
|  | ICAs use within 7 days of starting dialysis | 1 | 0 | 0 | **1** |  |
|  | Creatinine monitoring | 0 | 0 | 1 | **1** | *Late/absence of creatinine monitoring were associated with higher risk of UDI.* |
|  | Nephrology related hospital stays | 1 | 0 | 0 | **1** |  |
|  | KRT preparation (access type) | 0 | 0 | 1 | **1** | *Those with a record of a fistula or PD catheter procedures were less likely to have an UDI than those with other access types.* |
|  | CKD coded prior to KRT | 0 | 0 | 1 | **1** | *Better CKD coding in GP records is associated with a lower risk of UDI.* |
|  | Prescribed statins in the 6 months prior to KRT | 0 | 0 | 1 | **1** | *Those who were prescribed statins had a reduced risk of UDI.* |
|  | GP consultations in the year prior to KRT | 0 | 0 | 1 | **1** | *More frequent GP consultation in the year prior to RRT was associated with a planned dialysis start.* |
|  | Hepatitis B vaccination ever prior to KRT | 0 | 1 | 1 | **1** | *Those who had the Hepatitis B vaccine prior to RRT start were at a lower risk of UDI.* |
|  | Number of physical disabilities | 0 | 0 | 1 | **1** | *The percentage of patients with physical disabilities was higher in areas/clusters with people at a lower risk of UDI.* |
|  | Stable inhabitants | 1 | 0 | 0 | **1** | *Those who stay longer at the same residence are more likely to have an UDI.* |
|  | Living in an urban (versus rural) location | 0 | 0 | 1 | **1** | *A decreased likelihood of UDI was found in patients living in urban settings.* |
|  | Attended private haemodialysis units | 0 | 0 | 1 | **1** |  |
|  | Cerebrovascular disease | 1 | 0 | 0 | **1** |  |
|  | Centre ESKD incidence | 1 | 0 | 0 | **1** | *The higher the incidence of ESKD in a given centre, the higher the likelihood of an individual starting dialysis as unplanned in that same centre.* |
|  | Previous myocardial infarction | 1 | 0 | 0 | **1** |  |
|  | Peripheral atherosclerosis | 1 | 0 | 0 | **1** |  |
|  | Life threatening problem | 1 | 0 | 0 | **1** |  |
|  | Rapid uraemia progression | 1 | 0 | 0 | **1** |  |
|  | First dialysis modality (HD versus PD) | 1 | 0 | 0 | **1** | *There was an association between starting on HD and starting as unplanned.* |
|  | Vascular access at initiation (CVC) | 1 | 0 | 0 | **1** | *Those whose vascular access at start of dialysis was a central venous catheter were more likely to have started as an emergency.* |
|  | Pre-dialysis anaemia management | 0 | 0 | 1 | **1** | *Unplanned starters were more likely to have a Hb measurement of < 10 g/dl prior to starting dialysis and less likely to be on ESA therapy.* |
|  | eGFR at first documented discussion of KRT | 0 | 0 | 1 | **1** | *Those with a higher eGFR (>8.0 ml/min/1.73 m2) at first documented discussion of KRT were less likely to start dialysis in an unplanned manner.* |
|  | Erythropoietin stimulating agent | 0 | 0 | 1 | **1** |  |
|  | Coronary artery disease | 1 | 0 | 0 | **1** |  |
|  | Year of incidence | 1 | 0 | 0 | **1** | *Increased year of incidence was associated with a higher likelihood of UDI.* |
|  | **PD POPULATIONS** |  |  |  |  |  |
|  | Previous HD | 1 | 0 | 0 | **1** |  |
|  | PD initiation after catheter implantation (days) | 1 | 0 | 0 | **1** |  |

**Summarising results from old and new studies**

Meta-analyses of cardiovascular disease and age, the two most common risk factors for unplanned dialysis identified in our review, are presented in Figure 2a and Figure 2b. These factors were also found to be important predictors of UDI by Hassan et al. Patients with cardiovascular disease were nearly twice as likely to initiate dialysis in an unplanned manner after combining the results from the original review and the updated review (pooled OR=1.85; 95% confidence interval (CI), 1.64 to 2.10, p < 0.001). Overall, there was weak evidence of heterogeneity in the data (I²=37.9%, p=0.106). It is worth noting that some studies reported odds ratios for the number of cardiovascular diseases compared with no disease, while others reported odds ratios for a single cardiovascular disease (e.g., myocardial infarction, stroke, or heart failure) separately, while others grouped several of these diseases. Further, some studies presented odds ratios for subcategories for UDI e.g., unplanned or urgent start with systemic illness, as opposed to broader groups of planned and unplanned.

The effect of age on UDI differed across studies (Figure 2b). In two of the larger studies which both used the French Registry data, younger patients were found to be at an increased risk of unplanned starts. This was different to the remaining studies included in the analysis which found older age had an association with UDI. Regarding the measurement of age, some studies reported effect estimates based on either a single year increase, or in age categories that were not consistent across studies. Further, definitions adopted for unplanned and planned dialysis initiation varied across studies. Thus, due to a high level of heterogeneity in the data, it was not appropriate to report the pooled estimate for this variable. In addition, the fact that studies were weighted by the precision of their respective effect estimates may not have been appropriate as the two largest studies had minimal influence in the pooled estimate in the analyses.

We observed heterogeneity in the data reported for the other common risk factors as well, such as albumin, haemoglobin, late referral, cause of kidney disease and cancer. In relation to referral time, what was classed as a late/early referral was not consistent. The time periods used to define late referral ranged anywhere from 1 to 12 months between the initial consultation to the date of dialysis initiation. It was also a challenge to pool the results from the two reviews as the recent studies often reported more advanced/adjusted analyses, while older studies presented more descriptive/unadjusted analyses: typically presenting the proportion of individuals in the unplanned and planned groups. Thus, an attempt was made to contact the authors to obtain the odds ratios where possible to aid comparisons, but this was unsuccessful in part because some of the studies in the initial review were undertaken more than ten years ago (*N=28*). Therefore, it was not possible to do the planned meta-analyses for many of the common risk factors or investigate the causes of heterogeneity among the study results.

**Figure 2a: A meta-analysis of cardiovascular disease as a risk factor for unplanned dialysis initiation.**

**
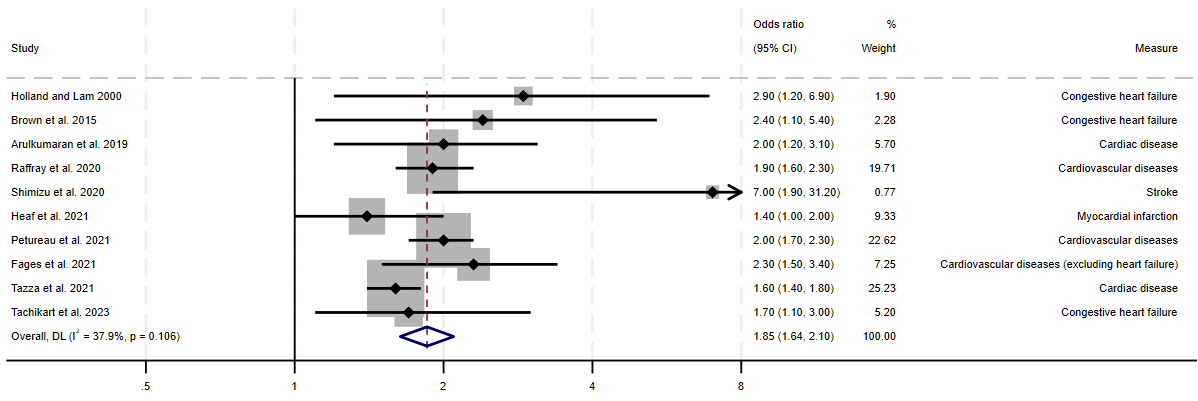
**

**Figure 2b: A meta-analysis of age as a risk factor for unplanned dialysis initiation**


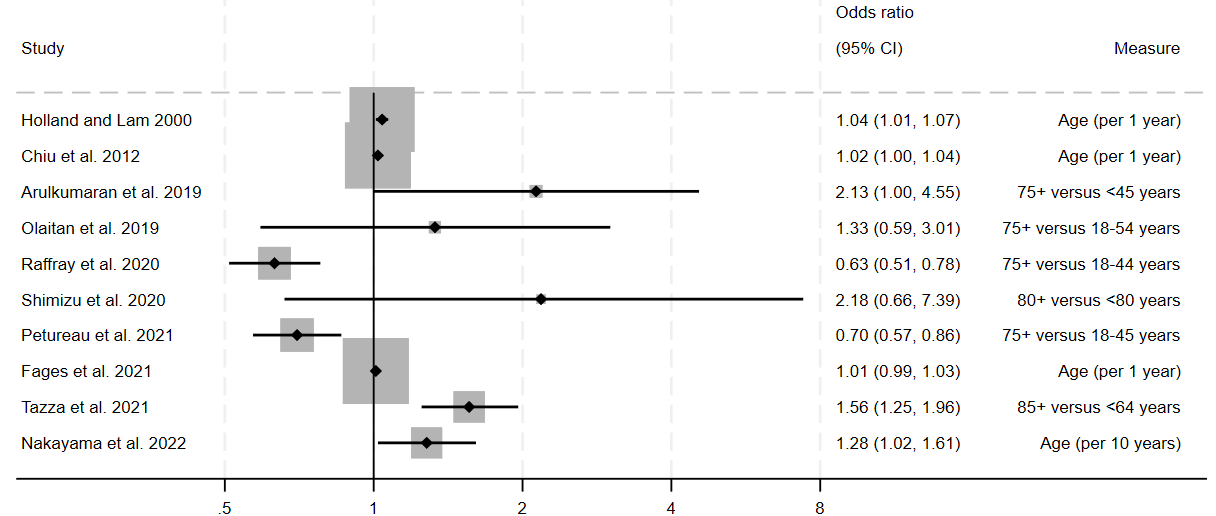


**Discussion**

In our updated review, we identified 17 international studies published between 2018 and 2023 that examined the risk factors for UDI. The most commonly reported predictors of an unplanned dialysis start were in line with those reported by Hassan et al. (2019) [3]. These included increased age, lower serum albumin, cardiovascular disease, cause of kidney disease, BMI, rate of eGFR decline and fewer nephrology visits prior to dialysis initiation. We also highlighted several other important factors associated with UDI, some of which are modifiable and have not been well studied, ranging from social factors to nephrology care. This information is useful for clinician as it may help identify patients most at risk of starting dialysis as unplanned, such as individuals who are living alone and are frail, allowing for better surveillance. By targeting high risk groups, clinicians may be able to educate patients about better management of their condition, reducing the likelihood of UDI. These interventions could lead to lower healthcare costs via a reduction in hospital stays and the resources allocated to certain ‘high risk’ individuals, as well as reducing mortality rates in the UDI population.

A retrospective cohort study in East London utilising primary care data linked to hospital audit data showed that the most significant modifiable risk factors for unplanned dialysis were the absence of a chronic kidney disease code in the GP record (OR 8.02, 95%CI 3.65 to 17.63) and the absence of prescribed lipid lowering medication (OR 2.37, 95%CI 1.05 to 5.34) [8]. The authors used the term ‘unplanned starters’ and ‘late referrals’ interchangeably, defining the outcome as individuals receiving less than 90 days follow-up by a nephrologist in a dedicated, multidisciplinary clinic for patients with an eGFR rate of less than 20mL/min/1.73m. Their findings were replicated in a recent study examining CKD coding practices and adverse outcomes [35]. In a large English CKD population, Cleary et al. (2022) showed a strong association between better CKD coding in GP records and fewer hospitalisation events, especially related to cardiovascular disease and heart failure. They found that coding practices varied the most for CKD stage 3a, suggesting areas of intervention to improve patient outcomes.

While most of the studies in our review were retrospective, a study by Fages et al (2021) [14] prospectively examined the risk factors for unplanned dialysis in over 3,000 patients in France, who were followed up from CKD to start of dialysis. They identified several clinical events , such as fluid overload, electrolytic disorders and acute-on-chronic kidney injury as common reasons for urgent dialysis start. They also highlighted novel risk factors like health literacy, living alone, hyper polypharmacy and inadequate pre-dialysis nephrology care, independent of age and the presence of heart failure. While previous studies have shown the importance of patient education and increased health literacy in reducing rates of UDI [2, 3], few have investigated the role of psychosocial factors, despite their potential impact on the effectiveness of pre-dialysis care pathways. Griva et al. (2019) [6] explored barriers related to patient-behaviour, time to access creation, and suboptimal dialysis initiation in a qualitative study in Singapore. They highlighted the importance of perceptual and emotional barriers which needed to be addressed to shift patients from avoidance and delay to timely access creation.

In another large-scale prospective study, Heaf et al. (2021) [27] investigated patient and centre level factors in the Baltic and Nordic countries, finding that late referral, cachexia, cardiovascular disease, hypalbuminaemia, and rapid uraemia progression were key factors in increasing the risk of suboptimal dialysis initiation (SDI). A strength of this study its clear outcome definition, based on catheter use for dialysis access, allowing for a better estimate of SDI incidence.

The issue of inconsistent definitions of UDI has been discussed at great length in the literature but does not seem to have been resolved [3, 36]. This heterogeneity presents challenges in estimating the incidence of unplanned dialysis starts and complicates data synthesis, making it difficult to compare risk factors across studies. The varying definitions may explain some of the opposing effects observed for age, sex and hypertension on UDI across studies. Many definitions in our review were based on vascular access type at dialysis initiation or urgent conditions under which dialysis started. Those with permanent access via an arteriovenous fistula (AVF) are typically described as optimal/planned starters, while individuals who start dialysis with a line/central venous catheter (CVC) are referred to as having a suboptimal initiation. Some authors considered the use of catheters (both tunnelled and non-tunnelled lines) as unplanned, while others adopted much more specific criteria, factoring in the initial and final access types, as well as considering the conditions they were under when they had their access put in [27]. The Renal Epidemiology and Information Network (REIN) definition, adopted by the French Registry, is considered the gold standard in France. However, the empirical 24-hour limit used in the definition may capture patients with similar characteristics to those starting dialysis under emergency conditions [34]. In the UK, the time between a patient first being seen by a nephrologist and commencement of RRT is often used to define an unplanned or urgent dialysis starts. Arulkumaran et al. (2019) described unplanned starters as patients known to UK renal services for more than 90 days who required acute RRT initiation, while urgent starters were those who were known for less than 90 days [12]. Similarly, Olaitan et al. (2019), defined unplanned dialysis as patients receiving less than 90 days follow-up by a nephrologist in a dedicated, multidisciplinary clinic, and those with an eGFR <20mL/min/1.73m² [8]. Due to the variability in the definitions used across studies and countries, caution is needed when comparing the reported risk factors for UDI.

Comparing Hassan et al.’s results with our updated review, we found significant differences in the quality of the studies. Newer studies were generally better designed, utilising adjusted or matched analyses, and larger and richer datasets, i.e. the seven French studies in our review used Registry data linked to hospital datasets. We also observed differences in study populations. Most included studies were based on HD patients, or a combination of HD and PD patients, with two of the studies focusing solely on PD populations ([28, 33]). These studies found no sociodemographic or clinical differences between the unplanned and planned PD groups, but the data were from small populations (<100 patients) in single-centre clinics in Brazil. Previous research suggests that outcomes for patients starting PD urgently are similar to those starting HD in the same manner. Lim et al. (2023) [9] showed that HD patients with a planned start had better early survival, but this was not the case for PD patients. Patient related barriers for unplanned or urgent dialysis initiation include (but are not limited to) the lack of operators who can place a peritoneal catheter within 48 hours.[37].

**Strengths and limitations**

A weakness of our study was the broad criteria used to define UDI. We included studies that described patients who are likely to be different from one another i.e. those that started dialysis acutely, in an ‘unplanned’ manner, urgently or in an emergency, and those that initiated dialysis with a CVC. It is therefore likely that the identified risk factors are not directly comparable due to the differences in these populations. This is further complicated by the fact that in some cases, vascular access at initiation of dialysis and late presentation were considered to be risk factors for UDI, while in others, these were the outcome of the study. Another limitation is the retrospective aspect of many of the included studies, making it more difficult to investigate several novel determinants of UDI and increasing potential information bias and confounding.

Despite these limitations, our study was strengthened by the inclusion of many large-scale studies utilising rich datasets in comparison to those reported by Hassan et al. Given that these high-quality studies often took advantage of the linkage of clinical and health care data at the patient level, the results reported can be more easily generalised to the wider populations.

**Conclusion**

Recent studies provide new insights into reasons for unplanned dialysis, some of which are modifiable, thus contribute to efforts in reducing the rate of UDI in the population. However, differences in clinical practices across the world in relation to how and when patients begin dialysis treatment, as well as varying definitions of UDI, tackling this issue remains a complex one. Contributing factors range from the availability of resources to patient education and preparedness, with no clear strategy for improving patient experience and outcomes. Future studies should focus on gathering evidence from populations with similar health systems to reduce the heterogeneity, as well as exploring the modifiable factors, such as psychosocial and social factors that have not been well studied. Additionally, future research should explore patient perspectives and the impact of unplanned dialysis initiation on quality of life. We hope that this can lead to more planned transitions to dialysis for patients with advanced CKD, and as a result, improve their outcomes and quality of life during this critical period.

**CONFLICT OF INTEREST STATEMENT**

All the authors declared no competing interests.

**ACKNOWLEDGEMENTS**

We would like to thank Sarah Herring for her assistance with developing the search strategy used in this systematic review.

**AUTHORS’ CONTRIBUTIONS**

W.M, K.B and S.S participated in the systematic review of the literature. F.C and Y.B.S provided clinical and epidemiological expertise on the subject. W.M undertook data analysis and all authors contributed to the interpretation of the results. W.M contributed to the writing and editing of the manuscript. W.M, K.B, S.S, F.C and Y.B.S participated in critical revision of the manuscript to its final form. All authors contributed to the conception and design of the study and read and approved the final manuscript.

**FUNDING**

This research received no specific grant from any funding agency in the public, commercial, or not-for-profit sectors.

**DATA AVAILABILITY STATEMENT**

The data underlying this article will be shared on reasonable request to the corresponding author.

**REFERENCES**

1. Evans, M. and K. Lopau, *The transition clinic in chronic kidney disease care.* Nephrol Dial Transplant, 2020. **35**(Suppl 2): p. ii4-ii10.

2. Machowska, A., et al., *Factors influencing access to education, decision making, and receipt of preferred dialysis modality in unplanned dialysis start patients.* Patient Prefer Adherence, 2016. **10**: p. 2229-2237.

3. Hassan, R., et al., *Risk Factors for Unplanned Dialysis Initiation: A Systematic Review of the Literature.* Can J Kidney Health Dis, 2019. **6**: p. 2054358119831684.

4. Weckmann, G.F.C., et al., *Diagnosis and management of non-dialysis chronic kidney disease in ambulatory care: a systematic review of clinical practice guidelines.* BMC Nephrol, 2018. **19**(1): p. 258.

5. Raffray, M., et al., *Predialysis Care Trajectories of Patients With ESKD Starting Dialysis in Emergency in France.* Kidney Int Rep, 2021. **6**(1): p. 156-167.

6. Griva, K., et al., *Patient-Related Barriers to Timely Dialysis Access Preparation: A Qualitative Study of the Perspectives of Patients, Family Members, and Health Care Providers.* Kidney Med, 2020. **2**(1): p. 29-41.

7. Kidney Disease: Improving Global Outcomes, C.K.D.W.G., *KDIGO 2024 Clinical Practice Guideline for the Evaluation and Management of Chronic Kidney Disease.* Kidney Int, 2024. **105**(4S): p. S117-S314.

8. Olaitan, A., et al., *Predictors of late presentation to renal dialysis: a cohort study of linked primary and secondary care records in East London.* BMJ Open, 2019. **9**(6): p. e028431.

9. Lim, J.H., et al., *The benefit of planned dialysis to early survival on hemodialysis versus peritoneal dialysis: a nationwide prospective multicenter study in Korea.* Sci Rep, 2023. **13**(1): p. 6049.

10. Molnar, A.O., et al., *Risk factors for unplanned and crash dialysis starts: a protocol for a systematic review and meta-analysis.* Syst Rev, 2016. **5**(1): p. 117.

11. Liao, C.T., et al., *Transitions of dialysis status and outcomes after the unplanned first dialysis: a nationwide population-based cohort study.* Sci Rep, 2023. **13**(1): p. 12867.

12. Arulkumaran, N., et al., *Causes and risk factors for acute dialysis initiation among patients with end-stage kidney disease-a large retrospective observational cohort study.* Clin Kidney J, 2019. **12**(4): p. 550-558.

13. Park, J.I., et al., *Not early referral but planned dialysis improves quality of life and depression in newly diagnosed end stage renal disease patients: a prospective cohort study in Korea.* PLoS One, 2015. **10**(2): p. e0117582.

14. Fages, V., et al., *Urgent-start dialysis in patients referred early to a nephrologist-the CKD-REIN prospective cohort study.* Nephrol Dial Transplant, 2021. **36**(8): p. 1500-1510.

15. Mendelssohn, D.C., et al., *Suboptimal initiation of dialysis with and without early referral to a nephrologist.* Nephrol Dial Transplant, 2011. **26**(9): p. 2959-65.

16. Saran, R., et al., *US Renal Data System 2016 Annual Data Report: Epidemiology of Kidney Disease in the United States.* Am J Kidney Dis, 2017. **69**(3 Suppl 1): p. A7-A8.

17. Page, M.J., et al., *The PRISMA 2020 statement: an updated guideline for reporting systematic reviews.* BMJ, 2021. **372**: p. n71.

18. Levey, A.S., et al., *Definition and classification of chronic kidney disease: a position statement from Kidney Disease: Improving Global Outcomes (KDIGO).* Kidney Int, 2005. **67**(6): p. 2089-100.

19. GA Wells, B.S., D O'Connell, J Peterson, V Welch, M Losos, P Tugwell, *The Newcastle-Ottawa Scale (NOS) for assessing the quality of nonrandomised studies in meta-analyses.*

20. Harris, R.J., et al., *Metan: Fixed- and Random-Effects Meta-Analysis.* The Stata Journal, 2008. **8**(1): p. 3-28.

21. Michel, A., et al., *Deleterious effects of dialysis emergency start, insights from the French REIN registry.* BMC Nephrol, 2018. **19**(1): p. 233.

22. Padilla, C.M., et al., *Geographic Variations in the Risk of Emergency First Dialysis for Patients with End Stage Renal Disease in the Bretagne Region, France.* Int J Environ Res Public Health, 2018. **16**(1).

23. Okaka, E.I., O.A. Adejumo, and A.A. Akinbodewa, *Late referral and associated factors among chronic kidney disease outpatients in Southern Nigeria.* Ann Afr Med, 2020. **19**(1): p. 47-52.

24. Petureau, A., et al., *Analysis of the association between emergency dialysis start in patients with end-stage kidney disease and non-steroidal anti-inflammatory drugs, proton-pump inhibitors, and iodinated contrast agents.* J Nephrol, 2021. **34**(5): p. 1711-1723.

25. Lee, T., et al., *Acute Kidney Injury before Dialysis Initiation Predicts Adverse Outcomes in Hemodialysis Patients.* Am J Nephrol, 2018. **47**(6): p. 427-434.

26. Shimizu, Y., et al., *Emergent initiation of dialysis is related to an increase in both mortality and medical costs.* Sci Rep, 2020. **10**(1): p. 19638.

27. Heaf, J., et al., *Suboptimal dialysis initiation is associated with comorbidities and uraemia progression rate but not with estimated glomerular filtration rate.* Clin Kidney J, 2021. **14**(3): p. 933-942.

28. Calice-Silva, V., et al., *Urgent vs. early-start peritoneal dialysis: patients' profile and outcomes.* J Bras Nefrol, 2021. **43**(1): p. 110-114.

29. Chung, E.Y., et al., *Factors associated with functional arteriovenous fistula at hemodialysis start and arteriovenous fistula non-use in a single-center cohort.* J Vasc Access, 2022. **23**(4): p. 558-566.

30. Tazza, L., et al., *Determinants of venous catheter hemodialysis onset and subsequent switch to arteriovenous fistula: An epidemiological study in Lazio region.* J Vasc Access, 2021. **22**(5): p. 749-758.

31. Nakayama, T., et al., *Effects of renin-angiotensin system inhibitors on the incidence of unplanned dialysis.* Hypertens Res, 2022. **45**(6): p. 1018-1027.

32. Takayama, S., et al., *Seasonal variation in emergent hemodialysis initiation in the late elderly: A single-center study.* Hemodial Int, 2022. **26**(2): p. 160-165.

33. Pilatti, M., et al., *Urgent vs. planned peritoneal dialysis initiation: complications and outcomes in the first year of therapy.* J Bras Nefrol, 2022. **44**(4): p. 482-489.

34. Tachikart, A., et al., *Determinants of urgent start dialysis in a chronic kidney disease cohort followed by nephrologists.* BMC Nephrol, 2023. **24**(1): p. 190.

35. Cleary, F., et al., *Association between practice coding of chronic kidney disease (CKD) in primary care and subsequent hospitalisations and death: a cohort analysis using national audit data.* BMJ Open, 2022. **12**(10): p. e064513.

36. Mendelssohn, D.C., C. Malmberg, and B. Hamandi, *An integrated review of "unplanned" dialysis initiation: reframing the terminology to "suboptimal" initiation.* BMC Nephrology, 2009. **10**(1): p. 22.

37. Chan, C.T., et al., *Dialysis initiation, modality choice, access, and prescription: conclusions from a Kidney Disease: Improving Global Outcomes (KDIGO) Controversies Conference.* Kidney Int, 2019. **96**(1): p. 37-47.

1. Studies could be excluded for multiple reasons, which is reflected in the numbers displayed in the figure. [↑](#footnote-ref-1)
